# Improving performance and generalizability in radiogenomics: a pilot study for prediction of IDH1/2 mutation status in gliomas with multicentric data

**DOI:** 10.1117/1.JMI.8.3.031905

**Published:** 2021-04-29

**Authors:** João Santinha, Celso Matos, Mário Figueiredo, Nikolaos Papanikolaou

**Affiliations:** a Clinical Computational Imaging Group, Champalimaud Research, Champalimaud Foundation, Lisboa, Portugal; b Universidade de Lisboa, Instituto de Telecomunicações, Instituto Superior Técnico, Lisboa, Portugal; c Champalimaud Clinical Center, Radiology Department, Champalimaud Foundation, Lisboa, Portugal

**Keywords:** radiogenomics, generalizability, multicentric, robust, invariance, IDH1/2 mutation status

## Abstract

**Purpose:** Radiogenomics offers a potential virtual and noninvasive biopsy. However, radiogenomics models often suffer from generalizability issues, which cause a performance degradation on unseen data. In MRI, differences in the sequence parameters, manufacturers, and scanners make this generalizability issue worse. Such image acquisition information may be used to define different environments and select robust and invariant radiomic features associated with the clinical outcome that should be included in radiomics/radiogenomics models.

**Approach:** We assessed 77 low-grade gliomas and glioblastomas multiform patients publicly available in TCGA and TCIA. Radiomics features were extracted from multiparametric MRI images (T1-weighted, contrast-enhanced T1-weighted, T2-weighted, and fluid-attenuated inversion recovery) and different regions-of-interest (enhancing tumor, nonenhancing tumor/necrosis, and edema). A method developed to find variables that are part of causal structures was used for feature selection and compared with an embedded feature selection approach commonly used in radiomics/radiogenomics studies, across two different scenarios: (1) leaving data from a center as an independent held-out test set and tuning the model with the data from the remaining centers and (2) use stratified partitioning to obtain the training and the held-out test sets.

**Results:** In scenario (1), the performance of the proposed methodology and the traditional embedded method was AUC: 0.75 [0.25; 1.00] versus 0.83 [0.50; 1.00], Sens.: 0.67 [0.20; 0.93] versus 0.67 [0.20; 0.93], Spec.: 0.75 [0.30; 0.95] versus 0.75 [0.30; 0.95], and MCC: 0.42 [0.19; 0.68] versus 0.42 [0.19; 0.68] for center 1 as the held-out test set. The performance of both methods for center 2 as the held-out test set was AUC: 0.64 [0.36; 0.91] versus 0.55 [0.27; 0.82], Sens.: 0.00 [0.00; 0.73] versus 0.00 [0.00; 0.73], Spec.: 0.82 [0.52; 0.94] versus 0.91 [0.62; 0.98], and MCC: −0.13 [−0.38;−0.04] versus −0.09 [−0.38;−0.02], whereas for center 3 was AUC: 0.80 [0.62; 0.95] versus 0.89 [0.56; 0.96], Sens.: 0.86 [0.48; 0.97] versus 0.86 [0.48; 0.97], Spec.: 0.72 [0.54; 0.85] versus 0.79 [0.61; 0.90], and MCC: 0.47 [0.41; 0.53] versus 0.55 [0.48; 0.60]. For center 4, the performance of both methods was AUC: 0.77 [0.51; 1.00] versus 0.75 [0.47; 0.97], Sens.: 0.53 [0.30; 0.75] versus 0.00 [0.00; 0.15], Spec.: 0.71 [0.35; 0.91] versus 0.86 [0.48; 0.97], and MCC: 0.23 [0.16; 0.31] versus. −0.32 [−0.46;−0.20]. In scenario (2), the performance of these methods was AUC: 0.89 [0.71; 1.00] versus 0.79 [0.58; 0.94], Sens.: 0.86 [0.80; 0.92] versus 0.43 [0.15; 0.74], Spec.: 0.87 [0.62; 0.96] versus 0.87 [0.62; 0.96], and MCC: 0.70 [0.60; 0.77] versus 0.33 [0.24; 0.42].

**Conclusions:** This proof-of-concept study demonstrated good performance by the proposed feature selection method in the majority of the studied scenarios, as it promotes robustness of features included in the models and the models’ generalizability by making used imaging data of different scanners or with sequence parameters.

## 1 Introduction

The discovery of associations between radiomics features and genomics characteristics or mechanisms, and the consequent development of prediction models based on imaging, is termed radiogenomics.^1^ This emerging subfield of radiomics is exceptionally appealing since radiomics provide a noninvasive, less expensive, and less time-consuming assessment of tumors, in comparison with genetic testing. Furthermore, its nondestructive assessment allows for multiple collections of information during the disease continuum. However, the assessment of the developed models using an independent held-out data set, or upon clinical deployment and validation, has demonstrated performance and generalizability issues. Some of these issues are related to data scarcity, population and prevalence shifts, and selection bias,^2^ which lead to the finding of nongeneralizable or spurious associations. In fact, it is desirable that the developed predictive models “work well” also for data from centers or settings that were not part of the training procedure.^3^^,^^4^

This study aims to demonstrate that a commonly used method for developing predictive models does not ensure generalizability and to show the potential of a recent method, developed to find causal relationships, for selecting robust and invariant (to distribution shifts caused by, e.g., different scanners, sequence parameters, or disease prevalence) features, leading to smaller and more generalizable models. As a use-case, we consider the development of a predictive model to determine the isocitrate dehydrogenase 1 and 2 (IDH1 and IDH2) mutation status from basic structural standard-of-care medical images^5^ and genomic data from TCIA/TCGA low-grade gliomas (LGG) and glioblastomas multiform (GBM) public archives.

The mutation status of both IDH1 and IDH2 (IDH1/2) genes is an important prognostic factor of both LGG and GBM,^6^ being since 2016 one of the principal genomic factors in the new World Health Organization Classification of Central Nervous System Tumors.^7^^–^^9^ In LGGs, IDH1/2 wild-types are characterized by poor, GBM-like prognosis, while the codeletion status of 1p and 19q chromosomes and G-CIMP subtypes offer additional stratification of the LGG IDH1/2 mutant and 1p/19q intact LGGs.^7^^–^^9^

As for GBMs, better prognosis has been found in the IDH1/2 mutant when compared with IDH1/2 wild-type GBMs. Besides prognosis, IDH1/2 was also demonstrated to provide information for treatment selection (temozolomide + radiation therapy is better than radiation therapy for wild-types, whereas no difference between treatments was found for mutants).^10^

Since the IDH1/2 mutation status is an important biomarker for patient stratification, prognosis, and treatment selection, we will demonstrate the use of the method developed by Peters et al.^3^ to improve robustness and generalizability of predictive models in the context of radiogenomics models to predict this genomic characteristic and compare it with a commonly used feature selection approach.

## 2 Methods

### 2.1 Study Population and Magnetic Resonance Imaging Data

This retrospective study used data from the cancer genome atlas glioblastoma multiform (TCGA-GBM)^11^ and low-grade glioma (TCGA-LGG)^12^ with available imaging data on the cancer imaging archive (TCIA).^13^^,^^14^ A total of 77 glioma patients with preoperative T1-weighted (T1w), contrast-enhanced T1-weighted (cT1w), fluid-attenuated inversion recovery (FLAIR), and T2-weighted (T2w) magnetic resonance (MR) images, IDH1/2 mutation status, and segmentation on the original image resolutions and orientations, for all four image types, were considered. The segmentations^15^ comprised three different regions:

• enhanced tumor
• nonenhanced tumor and necrosis
• edema.

**Fig. 1:**
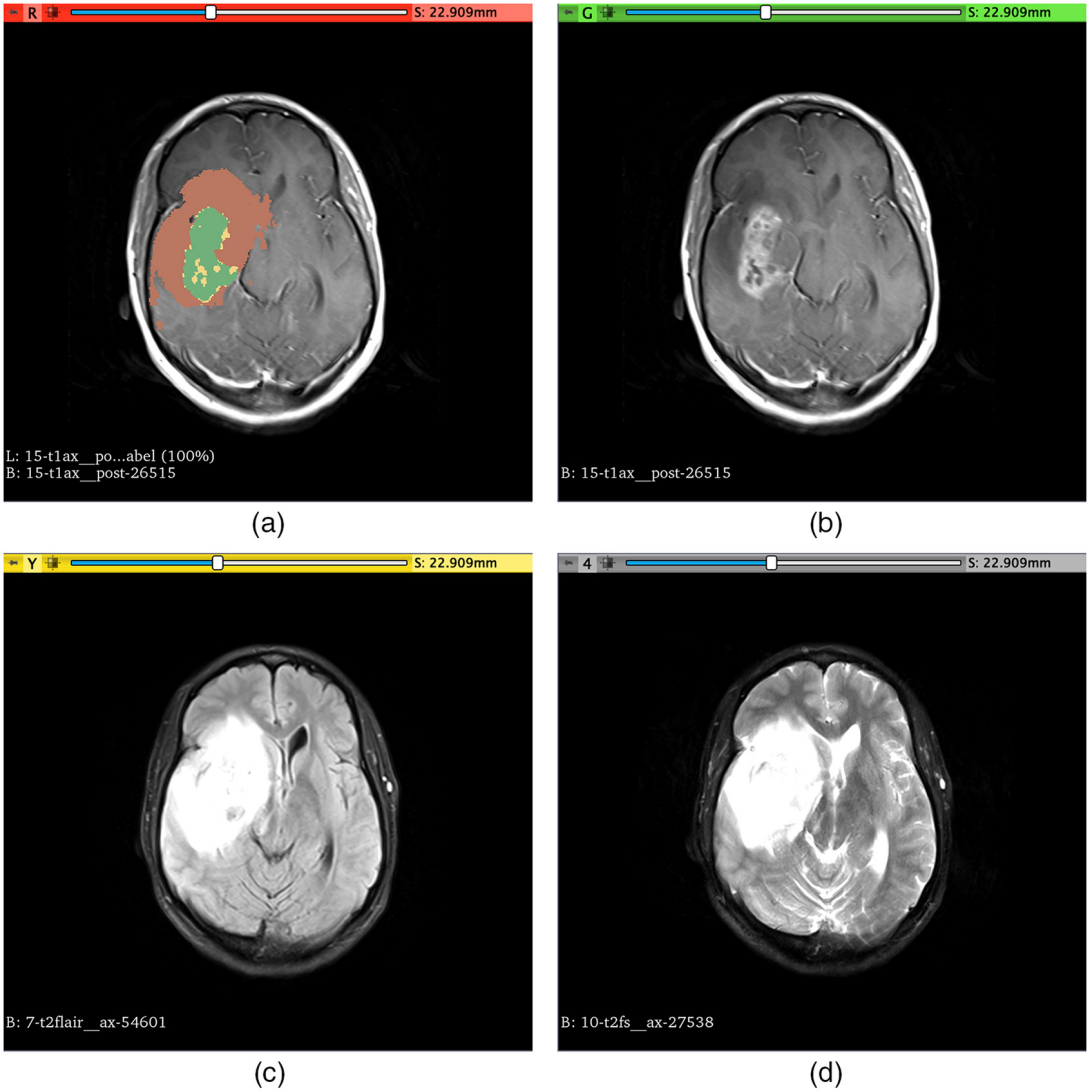
Example of segmentation of the different regions used to extract the radiomic features (TCIA case ID – TCGA-14-3477) overlaid in the cT1w image (a). Enhanced tumor region (L1) is shown in green, nonenhanced tumor and necrosis (L2) are shown in yellow, and edema (L3) is shown in brown. (b) The cT1w image without segmentation overlay; (c) FLAIR image; (d) T2w image.

An example of the segmentation of these regions is shown in Fig. 1.

Of these 77 glioma patients, 23 gliomas were classified as grade II, 35 as grade III, and 17 as grade IV. As for the IDH1/2 mutation status, 51 gliomas presented IDH1/2 mutation, and the remaining 26 gliomas had IDH1/2 wild-type. These data came from four different hospitals, each providing a different number of cases and IDH1/2 mutation prevalence. A more detailed description of the number of cases provided by each hospital for each class (IDH1/2 mutant and IDH1/2 wild-type) and the corresponding grade prevalence is shown in Table 1. For future reference, in this study, each of the hospitals will be referred to by the Center ID provided in Table 1.

**Table 1:** List of hospitals and corresponding total number of cases and number of IDH1/2 mutated and wild-type cases with respective tumor grades (II, III, and IV).

Hospital (center ID)	No. of cases	No. of IDH1/2 mutant (grades)	No. of IDH1/2 wild-type (grades)
Case Western (1)	7	4 (4 – II)	3 (1 – III; 2 – IV)
Case Western – St. Joes (2)	12	11 (5 – II; 6 – III)	1 (1 – II)
Henry Ford Hospital (3)	36	29 (12 – II; 15 – III; 2 – IV)	7 (5 – III; 2 – IV)
Thomas Jefferson University (4)	22	7 (2 – II; 5 – III)	15 (1 – II; 3 – III; 11 – IV)

### 2.2 Feature Extraction

Image preprocessing and feature extraction were performed using pyradiomics [version 2.2.0 (available in a Github repository: https://github.com/Radiomics/pyradiomics/tree/2.2.0)].^16^ Images with isotropic voxel dimensions ($1\times 1\times 1\;{\mathrm{mm}}^{3}$) and corresponding segmentations were made available in the context of the brain tumor segmentation challenges.^17^ However, a considerable part of the original images is not isotropic (some have 5/6  mm slice thickness), and image upsampling is not advisable for feature extraction as this may create nonacquired information and is dependent on the interpolation method. For this reason, the original images and a two-dimensional feature extraction with downsampling of the in-plane resolution of the images to the lowest in-plane resolution across all images were used, making use of pyradiomics preprocessing functionalities. Before feature extraction, pyradiomics intensity normalization was used to ensure that all images had a mean value of 300 and a standard deviation (s.d.) of 100, which under a standard distribution of intensity values would have the majority of values between 0 and 600 (mean ±3 s.d.). The different segmentation labels drawn on the T1w, cT1w, FLAIR, and T2w images comprising enhanced tumor, nonenhanced tumor and necrosis, and edema regions were used for the feature extraction. Features from class shape, first-order, gray-level co-occurrence matrix (GLCM), gray-level run length matrix (GLRLM), gray-level size zone matrix (GLSZM), neighboring gray tone difference matrix (NGTDM), and gray-level dependence matrix (GLDM) were extracted from the original images and filtered images (Laplacian of Gaussian, LoG, with σ={1.015,2,3}  mm; wavelet – two levels) using pyradiomics. Fixed bin-width was used following the results presented in Refs. 18 and 19, and the value was chosen following the pyradiomics documentation. A summary of the feature extraction parameters is provided in Table 2. The parameter files used for the feature extraction are available in a Github repositoty: https://github.com/JoaoSantinha/Radiogenomics_Gliomas_Pilot. A total of 12,456 radiomic features were extracted for each patient and used for feature selection and model development.

**Table 2:** List of feature extraction parameters used for each MRI sequence.

Feature extraction parameters	cT1w	T1w	T2w	FLAIR
Normalization scale	100	100	100	100
Voxel array shift	300	300	300	300
Resampled pixel spacing (mm)	1.016×1.016	1.016×1.016	1.016×1.016	1.016×1.016
Resegment range (mode: sigma)	[−3,3]	[−3,3]	[−3,3]	[−3,3]
Bin width	5	2	5	5
LoG sigma (mm)	[1.016, 2, 3]	[1.016, 2, 3]	[1.016, 2, 3]	[1.016, 2, 3]
Wavelet number of levels	2	2	2	2

To allow for the differentiation of features extracted from different image sequences and tumor regions, we first appended to each feature name the image sequence followed by the suffix “L1,” “L2,” or “L3,” corresponding to enhanced tumor, nonenhanced tumor and necrosis, or edema, respectively.

### 2.3 Feature Selection and Classification

As a high-dimensional problem, where the number of features greatly exceeds the number of cases, feature selection is needed to train a model with good performance and generalizable. However, in these cases, a nonunique solution may be obtained, leading to loss of generalizability. In addition, this poor generalizability of models developed for MRI may also be attributed to the nonquantitative nature of specific sequences, sequence differences between vendors, and a large number of tunable sequence parameters. Therefore, a feature selection method capable of mitigating these issues is paramount to ensuring good generalizability.

One of the approaches widely adopted to perform feature selection is the use of embedded methods, such as the least absolute shrinkage and selection operator (lasso).^20^ More recently, Peters et al.^3^ suggested a method to find predictors in the context of causal inference. Their method selects variables that are part of causal structures on the basis that they should remain robust and invariant to changes in environments (different observation settings or interventions). In our context, texture features may reflect the IDH1/2 mutation status but not the other way around, and the different environments can be seen as different MRI scanners or sequences with different parameters, as found in multicentric studies. Following this, the method from Peters et al. will be used to select features that are robust and show invariant relationships with the IDH1/2 mutation status, despite the changes in image acquisition environments, and that may improve models performance and generalizability across the set of nonobserved environments, which is much larger than the set of observed environments used to find these features and tune the model.

The proposed approach includes several preselection methods to constrain the search space. In this study, we will assess lasso-based and stability-based feature preselection. The features showing robustness and invariance will be used in a logistic regression model with lasso regularization, where the regularization strength, λ, will be optimized. Both approaches will be compared with logistic regression with lasso regularization using all radiomic features, a very common approach in radiomics/radiogenomics studies.

### 2.4 Classification Evaluation

The different methods will be assessed and compared using two different scenarios:

1. Data from three centers are used to tune the models and data from the fourth center are used as an independent test set.
2. Data from all four centers are used to perform stratified partitioning based on the IDH1/2 mutation status prevalence to tune the model on 70% of data, and the remaining 30% are used to assess the performance of models.

The regularization strength λ was optimized based on 10 times repeated threefold cross-validation, using as the optimization metric the Matthews correlation coefficient (MCC),^21^ which is independent of the class balance and not affected by choice of positive class, such as the accuracy and F1-score.^22^ The area under the receiver operating characteristic curve (AUC), accuracy, sensitivity, and specificity performance metrics were also obtained for each model and scenario under study. The 95% confidence intervals (CIs) for the performance metrics on the test sets were determined using bootstrapping with 2000 replications for the AUC and the Wilson score interval method for the remaining metrics. If the 95% CI for the AUC includes 0.5, there is no support to claim that the method outperforms random guessing with a significance level of 5%. Stratified bootstrapping with 2000 replications was used to compare test AUCs of models obtained using the different feature selection methods. Multiple comparison correction was applied using the Holm–Bonferroni method, as each model was compared multiple times. The no information rate (NIR) was also determined and compared with the accuracy using a one-sided binomial test. The level of significance, α, was set to 5%. Confusion matrices were also produced for the different test data sets in scenario 1 and for the test data set in scenario 2.

## 3 Results

In this section, we demonstrate the use of the robust and invariant features selected through the proposed methodology and compare them with a commonly used approach. The section is divided into two subsections, Secs. 3.1 and 3.2, reflecting the different evaluation scenarios. Triplets of the models, comprising lasso preselected robust and invariant features model (A1), stability preselected robust and invariant features model (A2), and all-features model (B), are tuned for the assessment and comparison in the different scenarios.

### 3.1 Training Using Data from Three Centers and Assessment on the Held-Out Center

The full data set comprises data from four different centers, with each of these four centers used once as the held-out test set and the remaining used to tune the models.

As shown in Table 3, models A1 and A2 showed better (p-values<0.001) cross-validation performance than model B in all cases, with AUCs of both models yielding statistically significant differences with model B, despite the overlap between the proposed models and model B when center 4 was used as the held-out test set. The p-values of the differences between cross-validation AUCs of models A1 and A2 were 0.006, 0.003, 0.055, and 0.195 for centers 1, 2, 3, and 4 as the held-out test sets, respectively. When using centers 1 or 2 as held-out test sets, the chosen optimization metric, MCC, was higher for model A1, whereas in the case of utilizing centers 3 or 4, MCC was higher for model A2, despite the considerable overlap between the models’ CIs. The performance of the different models in the held-out test sets is also shown in Table 3, and the corresponding confusion matrices are aggregated into Table 4.

**Table 3:** Performance metrics of the different models, A1, A2, and B, when trained with the different combinations of three out of the four centers and tested on the held-out center. AUC, area under the receiver operating characteristic curve; Acc, accuracy; Sens, sensitivity; Spec, specificity; MCC, Matthews correlation coefficient.

		Model A1		Model A2		Model B	
		CV [95% CI]	Test [95% CI]	CV [95% CI]	Test [95% CI]	CV [95% CI]	Test [95% CI]
Train with centers 2, 3, and 4; test with center 1	AUC	0.95 [0.94; 0.97]	0.75 [0.25; 1.00]	0.93 [0.91; 0.95]	0.67 [0.25; 1.00]	0.81 [0.78; 0.84]	0.83 [0.50; 1.00]
	Acc	0.90 [0.88; 0.92]	0.71 [0.35; 0.91]	0.89 [0.86; 0.91]	0.71 [0.35; 0.91]	0.75 [0.72; 0.78]	0.71 [0.35; 0.91]
	Sens	0.84 [0.78; 0.90]	0.67 [0.20; 0.93]	0.86 [0.80; 0.92]	0.67 [0.20; 0.93]	0.55 [0.45; 0.65]	0.67 [0.20; 0.93]
	Spec	0.93 [0.91; 0.95]	0.75 [0.30; 0.95]	0.90 [0.87; 0.93]	0.75 [0.30; 0.95]	0.85 [0.82; 0.88]	0.75 [0.30; 0.95]
	MCC	0.77 [0.72; 0.82]	0.42 [0.19; 0.68]	0.75 [0.70; 0.81]	0.42 [0.19; 0.68]	0.42 [0.33; 0.51]	0.42 [0.19; 0.68]
Train with centers 1, 3, and 4; test with center 2	AUC	0.92 [0.90; 0.94]	0.64 [0.36; 0.91]	0.91 [0.89; 0.94]	0.82 [0.55; 1.00]	0.84 [0.81; 0.86]	0.55 [0.27; 0.82]
	Acc	0.85 [0.82; 0.87]	0.75 [0.46; 0.91]	0.85 [0.82; 0.87]	0.67 [0.39; 0.86]	0.77 [0.75; 0.80]	0.83 [0.55; 0.95]
	Sens	0.79 [0.75; 0.83]	0.00 [0.00; 0.73]	0.76 [0.69; 0.83]	0.00 [0.00; 0.73]	0.59 [0.53; 0.65]	0.00 [0.00; 0.73]
	Spec	0.87 [0.84; 0.91]	0.82 [0.52; 0.94]	0.89 [0.87; 0.91]	0.73 [0.43; 0.90]	0.87 [0.84; 0.90]	0.91 [0.62; 0.98]
	MCC	0.67 [0.61; 0.72]	-0.13 [-0.38; -0.04]	0.66 [0.60; 0.72]	-0.17 [-0.41; -0.06]	0.48 [0.42; 0.54]	-0.09 [-0.38; -0.02]
Train with centers 1, 2, and 4; test with center 3	AUC	0.91 [0.89; 0.93]	0.80 [0.62; 0.95]	0.91 [0.89; 0.93]	0.85 [0.38; 0.86]	0.83 [0.80; 0.86]	0.89 [0.56; 0.96]
	Acc	0.84 [0.80; 0.88]	0.75 [0.58; 0.86]	0.85 [0.83; 0.88]	0.78 [0.61; 0.88]	0.76 [0.72; 0.79]	0.81 [0.64; 0.90]
	Sens	0.75 [0.66; 0.83]	0.86 [0.48; 0.97]	0.82 [0.74; 0.89]	0.57 [0.25; 0.84]	0.54 [0.44; 0.64]	0.86 [0.48; 0.97]
	Spec	0.88 [0.84; 0.93]	0.72 [0.54; 0.85]	0.87 [0.83; 0.91]	0.83 [0.65; 0.92]	0.87 [0.82; 0.91]	0.79 [0.61; 0.90]
	MCC	0.64 [0.55; 0.73]	0.47 [0.41; 0.53]	0.69 [0.63; 0.74]	0.36 [0.30; 0.42]	0.44 [0.34; 0.54]	0.55 [0.48; 0.60]
Train with centers 1, 2, and 3; test with center 4	AUC	0.87 [0.84; 0.89]	0.77 [0.51; 1.00]	0.84 [0.81; 0.87]	0.72 [0.44; 0.93]	0.82 [0.78; 0.85]	0.75 [0.47; 0.97]
	Acc	0.82 [0.79; 0.85]	0.59 [0.38; 0.76]	0.83 [0.80; 0.86]	0.36 [0.19; 0.57]	0.77 [0.74; 0.80]	0.27 [0.13; 0.48]
	Sens	0.59 [0.50; 0.67]	0.53 [0.30; 0.75]	0.62 [0.53; 0.71]	0.13 [0.03; 0.37]	0.56 [0.46; 0.65]	0.00 [0.00; 0.15]
	Spec	0.93 [0.90; 0.97]	0.71 [0.35; 0.91]	0.94 [0.91; 0.96]	0.86 [0.48; 0.97]	0.88 [0.84; 0.92]	0.86 [0.48; 0.97]
	MCC	0.59 [0.52; 0.66]	0.23 [0.16; 0.31]	0.61 [0.55; 0.68]	-0,01 [-0.07; 0.00]	0.47 [0.39; 0.55]	-0.32 [-0.46; -0.20]

**Table 4:** Confusion matrix of the different models, A1, A2, and B, on each of the corresponding independent held-out test sets (WT denotes wild-type).

			Reference	
		Prediction	Mutant	WT
Test using center 1	Model A1	Mutant	3	1
		WT	1	2
	Model A2	Mutant	3	1
		WT	1	2
	Model B	Mutant	3	1
		WT	1	2
Test using center 2	Model A1	Mutant	9	1
		WT	2	0
	Model A2	Mutant	8	1
		WT	3	0
	Model B	Mutant	10	1
		WT	1	0
Test using center 3	Model A1	Mutant	21	1
		WT	8	6
	Model A2	Mutant	24	3
		WT	5	4
	Model B	Mutant	23	1
		WT	6	6
Test using center 4	Model A1	Mutant	5	7
		WT	2	8
	Model A2	Mutant	6	13
		WT	1	2
	Model B	Mutant	6	15
		WT	1	0

When using center 1 as the independent held-out test set, all models showed equal performance (accuracy=0.71 [0.35; 0.91], sensitivity=0.67 [0.20; 0.93], specificity=0.75 [0.30; 0.95], MCC=0.42 [0.19; 0.68]), with the AUC being the only metric exhibiting differences, with model B having the highest AUC (0.83 [0.50; 1.00]). However, differences between the AUC of different models were not statistically significant (p-values>0.05). As the AUC of models A1 and A2 includes 0.5, claims of outperforming random guessing are not supported at the selected significance level. With center 2 as the independent held-out test set, it is possible to observe in Tables 3 and 4 that all models misclassified the unique wild-type case present in this data set. In terms of specificity, model B appeared to perform better (0.91 [0.62; 0.98]), followed by model A1 (0.82 [0.52; 0.94]) and model A2 (0.73 [0.43; 0.90]), but the overlap between CIs does not grant statistical significance to these differences. The assessment on the data from center 3 showed that both models A1 and B yielded equal sensitivity (0.86 [0.48; 0.97]), whereas model B demonstrated higher specificity than A1 (0.79 [0.61; 0.90] versus 0.72 [0.54; 0.85]). As for model A2, it provided higher specificity (0.83 [0.65; 0.92]) than models A1 and B, but with a considerably lower sensitivity (0.57 [0.25; 0.84]). Despite the differences in sensitivity and specificity, the corresponding CIs of the different models presented a considerable overlap. In the latter case, where data from center 4 was used as the independent held-out test set, despite models A2 and B showing higher specificity than A1 (0.86 [0.48; 0.97] versus 0.71 [0.35; 0.91]), their sensitivity was lower (0.13 [0.03; 0.37] versus 0.00 [0.00; 0.15] versus 0.53 [0.30; 0.75]). Nonetheless, for the test sets, only the sensitivity and MCC of center 4 for model A1 yielded CIs higher than that of model B.

**Fig. 2:**
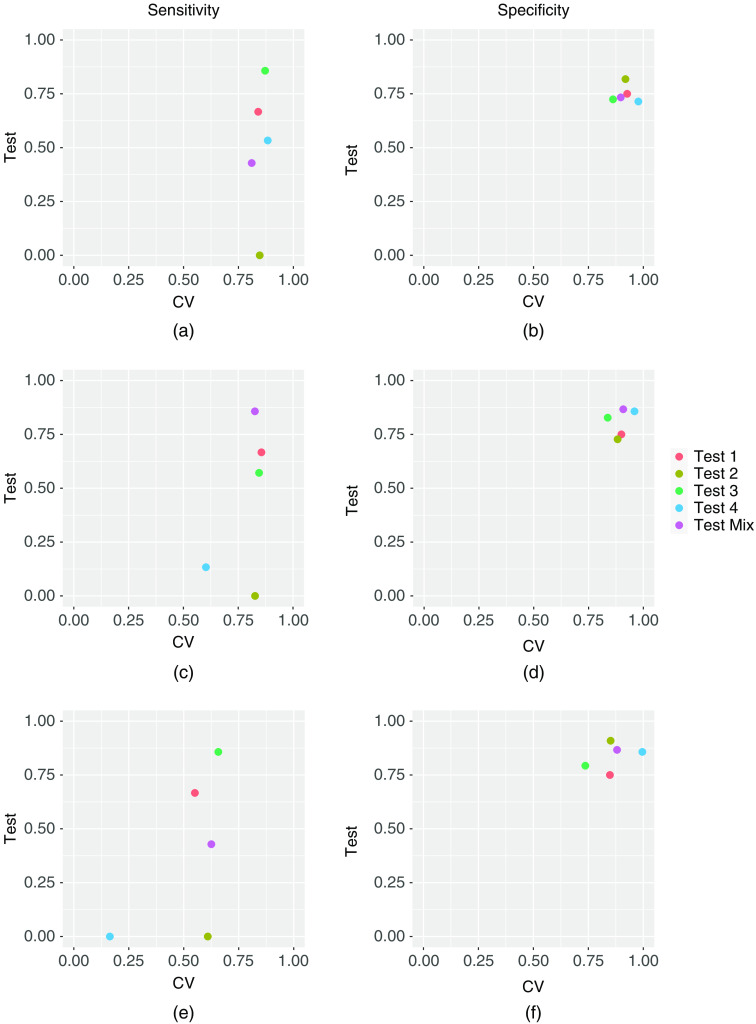
Sensitivity and specificity of cross-validation versus testing for different cases. (a), (b) Sensitivity and specificity of model A1;(c), (d) sensitivity and specificity of model A2; (e), (f) sensitivity and specificity of model B.

A visual comparison of cross-validation and test sensitivity and specificity performances, for the different models in each of the cases studied, is shown in Fig. 2. The three models’ sensitivity and specificity for the second scenario, presented in Sec. 3.2, are also included in this figure.

### 3.2 Full Data Set Stratified Partitioning into Training and Held-Out Test Sets

In this second scenario, the full data set, comprising data from all four centers, was split into a training set and a held-out test set, using stratified partitioning based on IDH1/2 mutation status, with 70% of the data used for training and 30% for test.

The cross-validation and test performance of the different models are shown in Table 5. Similar to the previous scenario, the AUC of models A1 and A2 were statistically superior (p-values<0.001) to model B, but statistical differences between models A1 and A2 were not found (p-value=0.57). Based on the optimization metric chosen, MCC, models A1 and A2 showed better performance than model B (0.77 [0.72; 0.82] versus 0.75 [0.70; 0.81] versus 0.42 [0.33; 0.51]). Other performance metrics were very similar between models A1 and A2 (accuracy: 0.90 [0.88; 0.92] versus 0.89 [0.86; 0.91]; sensitivity: 0.84 [0.78; 0.90] versus 0.86 [0.80; 0.92]; and specificity: 0.93 [0.91; 0.95] versus 0.90 [0.87; 0.93]). On the held-out test set, model A2 was able to yield a performance within the 95% CI attained in the cross-validation across all metrics, with the exception of AUC, as shown in Table 5. Model A1 showed a considerable drop in all performance metrics, whereas model B showed a slight decrease in all metrics except for specificity (CV – 0.85 [0.82; 0.88] versus test −0.87 [0.62; 0.96]). The corresponding confusion matrices of models A1, A2, and B are shown in Table 6.

**Table 5:** Performance metrics of the different models, A1, A2, and B, on the cross-validation (CV) and the held-out test (Test) set, corresponding to 30% of the whole data set and obtained through stratified partitioning based on IDH1/2 mutation status. AUC, area under the receiver operating characteristic curve; Acc, accuracy; Sens, sensitivity; Spec, specificity; MCC, Matthews correlation coefficient; CI, confidence interval.

	Model A1		Model A2		Model B	
	CV [95% CI]	Test [95% CI]	CV [95% CI]	Test [95% CI]	CV [95% CI]	Test [95% CI]
AUC	0.95 [0.94; 0.97]	0.80 [0.58; 0.95]	0.93 [0.91; 0.95]	0.89 [0.71; 1.00]	0.81 [0.78; 0.84]	0.79 [0.58; 0.94]
Acc	0.90 [0.88; 0.92]	0.64 [0.42; 0.80]	0.89 [0.86; 0.91]	0.86 [0.66; 0.95]	0.75 [0.72; 0.78]	0.73 [0.51; 0.86]
Sens	0.84 [0.78; 0.90]	0.43 [0.15; 0.74]	0.86 [0.80; 0.92]	0.86 [0.48; 0.97]	0.55 [0.45; 0.65]	0.43 [0.15; 0.74]
Spec	0.93 [0.91; 0.95]	0.73 [0.48; 0.89]	0.90 [0.87; 0.93]	0.87 [0.62; 0.96]	0.85 [0.82; 0.88]	0.87 [0.62; 0.96]
MCC	0.77 [0.72; 0.82]	0.16 [0.10; 0.24]	0.75 [0.70; 0.81]	0.70 [0.60; 0.77]	0.42 [0.33; 0.51]	0.33 [0.24; 0.42]

**Table 6:** Confusion matrix of the different models, A1, A2, and B, on held-out test set.

	Prediction	Reference	
		Mutant	WT
Model A1	Mutant	11	4
	WT	4	3
Model A2	Mutant	13	1
	WT	2	6
Model B	Mutant	13	4
	WT	2	3

For both scenarios, the models’ accuracies on test sets were compared with the NIR, and the p-values are provided in Table 7. For scenario 1, these comparisons did not show significantly greater accuracy than the NIR, whereas model A2 showed accuracy significantly greater than the NIR for scenario 2.

**Table 7:** P-values of one-sided tests to assess if accuracy was greater than the NIR.

	p-value [Acc > NIR]		
	Model A1	Model A2	Model B
Test 1	0.359	0.359	0.359
Test 2	0.986	0.998	0.928
Test 3	0.854	0.745	0.599
Test 4	0.873	1.000	1.000
Mix	0.758	0.048	0.420

**Fig. 3:**
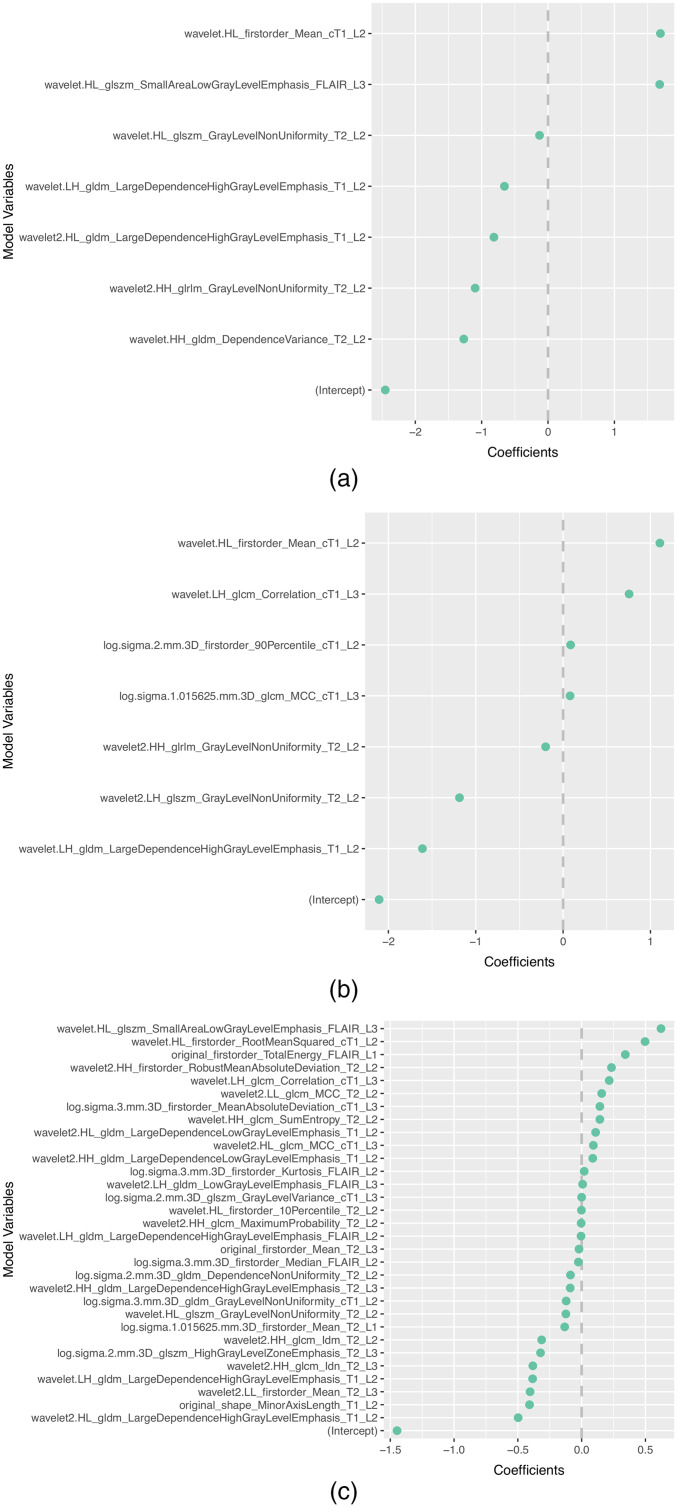
Representation of coefficients of selected features for models (a) A1, (b) A2, and (3) B when tuned with data from centers 2, 3, and 4.

**Fig. 4:**
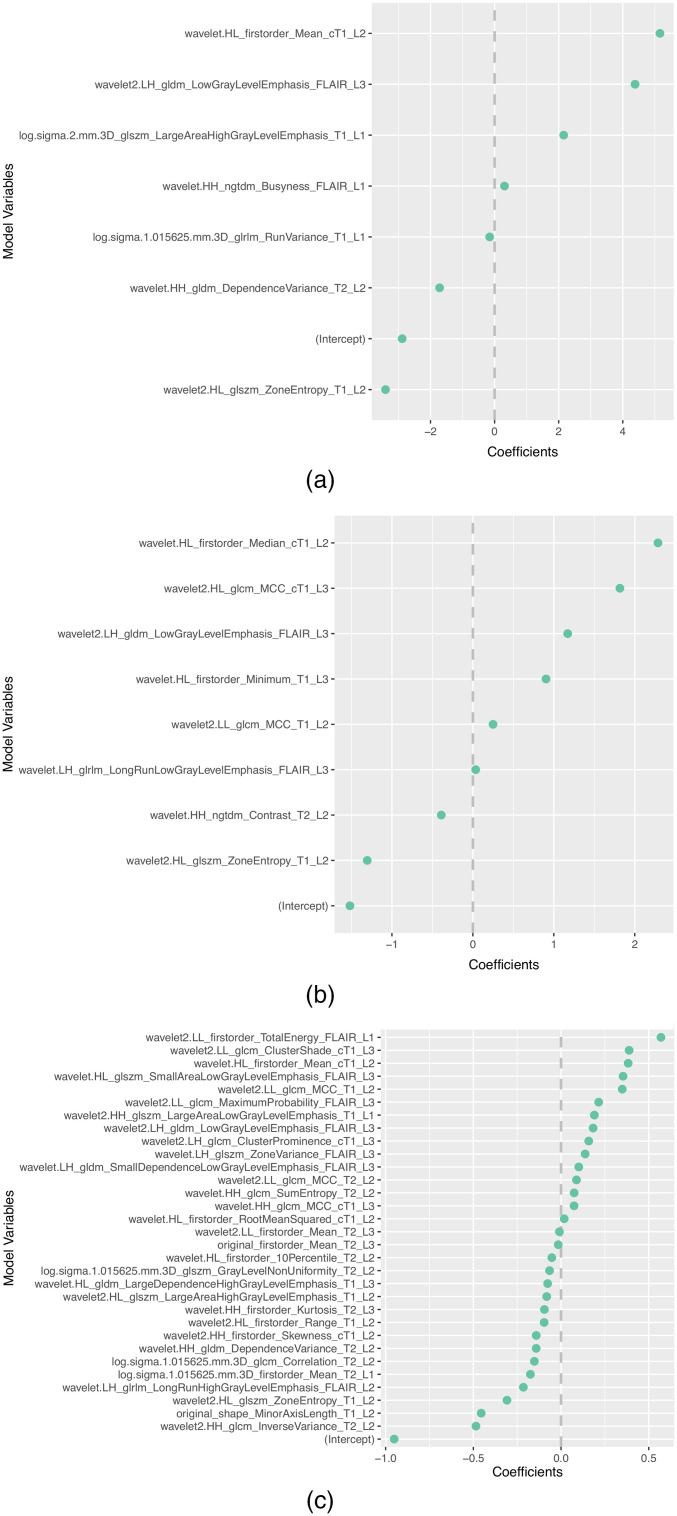
Representation of coefficients of selected features for models (a) A1, (b) A2, and (c) B when tuned with data from centers 1, 3, and 4.

**Fig. 5:**
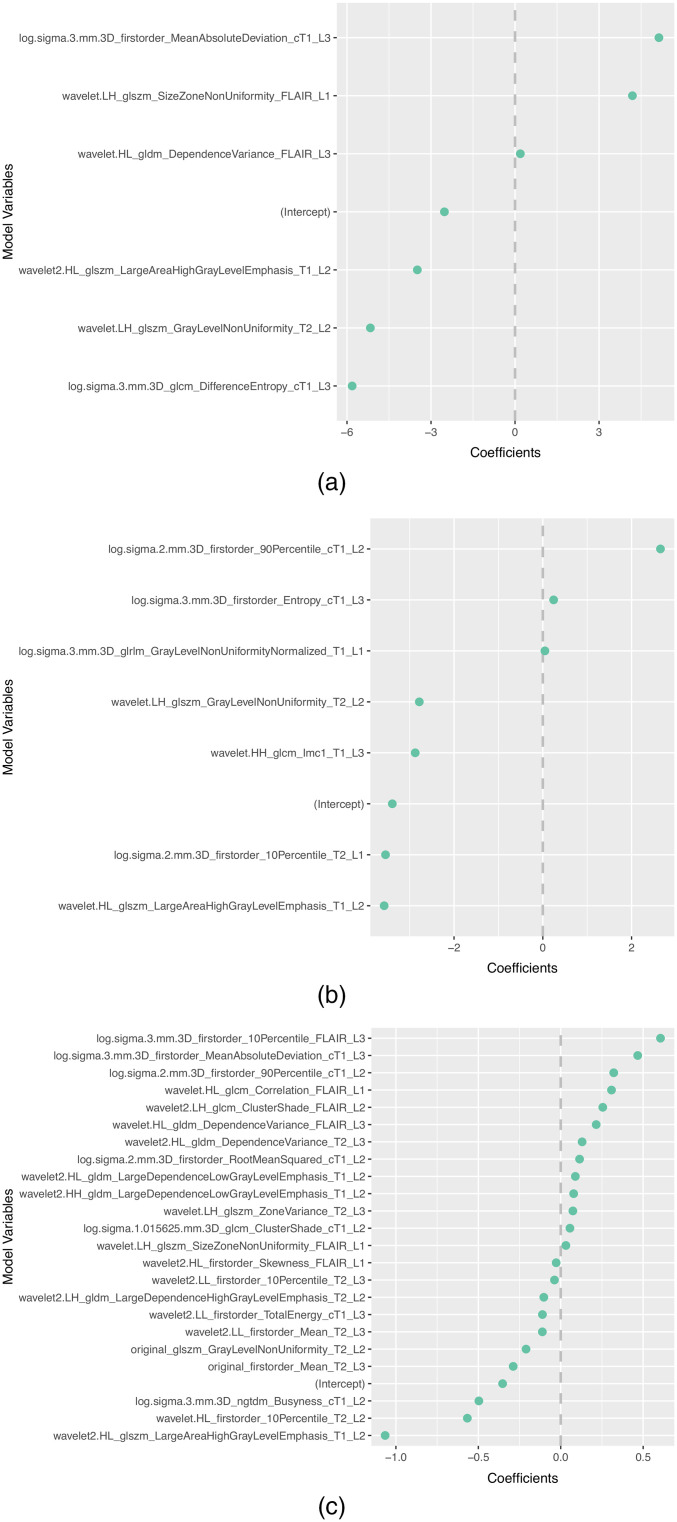
Representation of coefficients of selected features for models (a) A1, (b) A2, and (c) B when tuned with data from centers 1, 2, and 4.

**Fig. 6:**
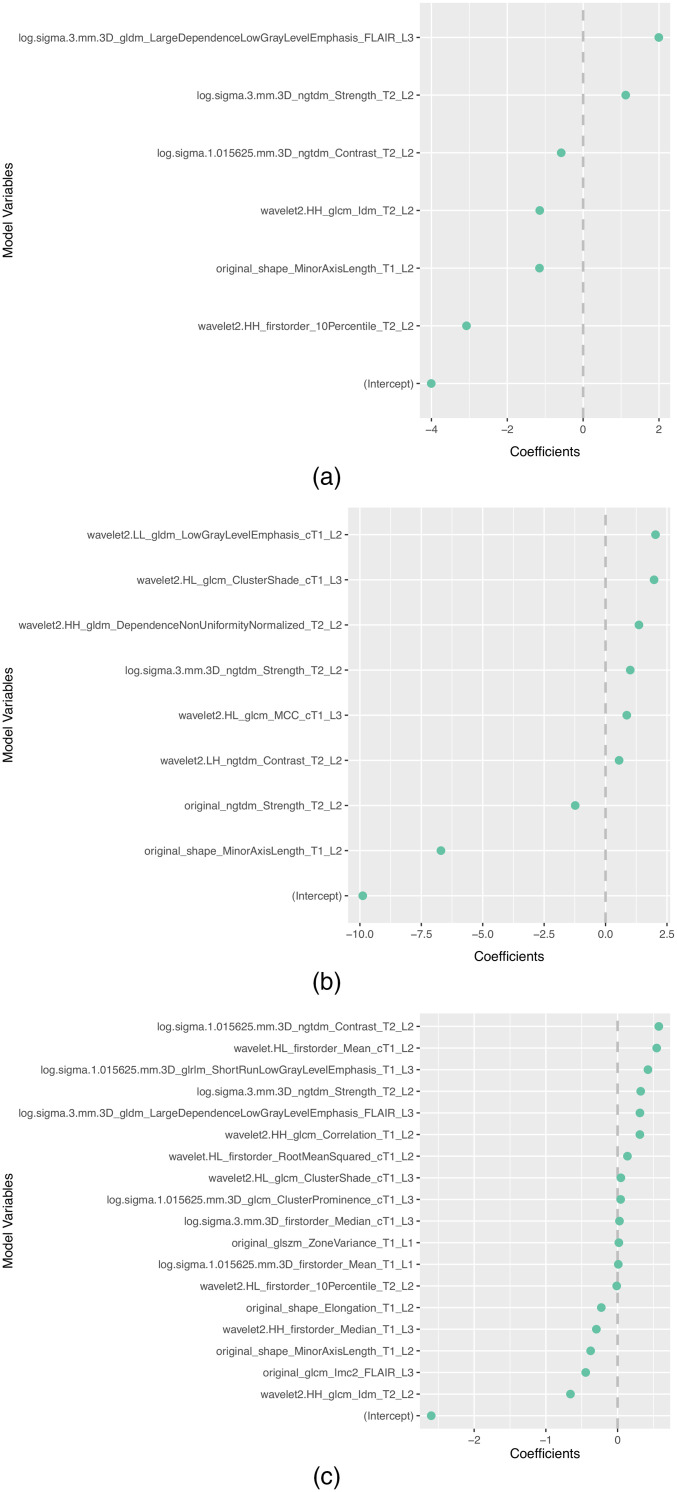
Representation of coefficients of selected features for models (a) A1, (b) A2, and (c) B when tuned with data from centers 1, 2, and 3.

**Fig. 7:**
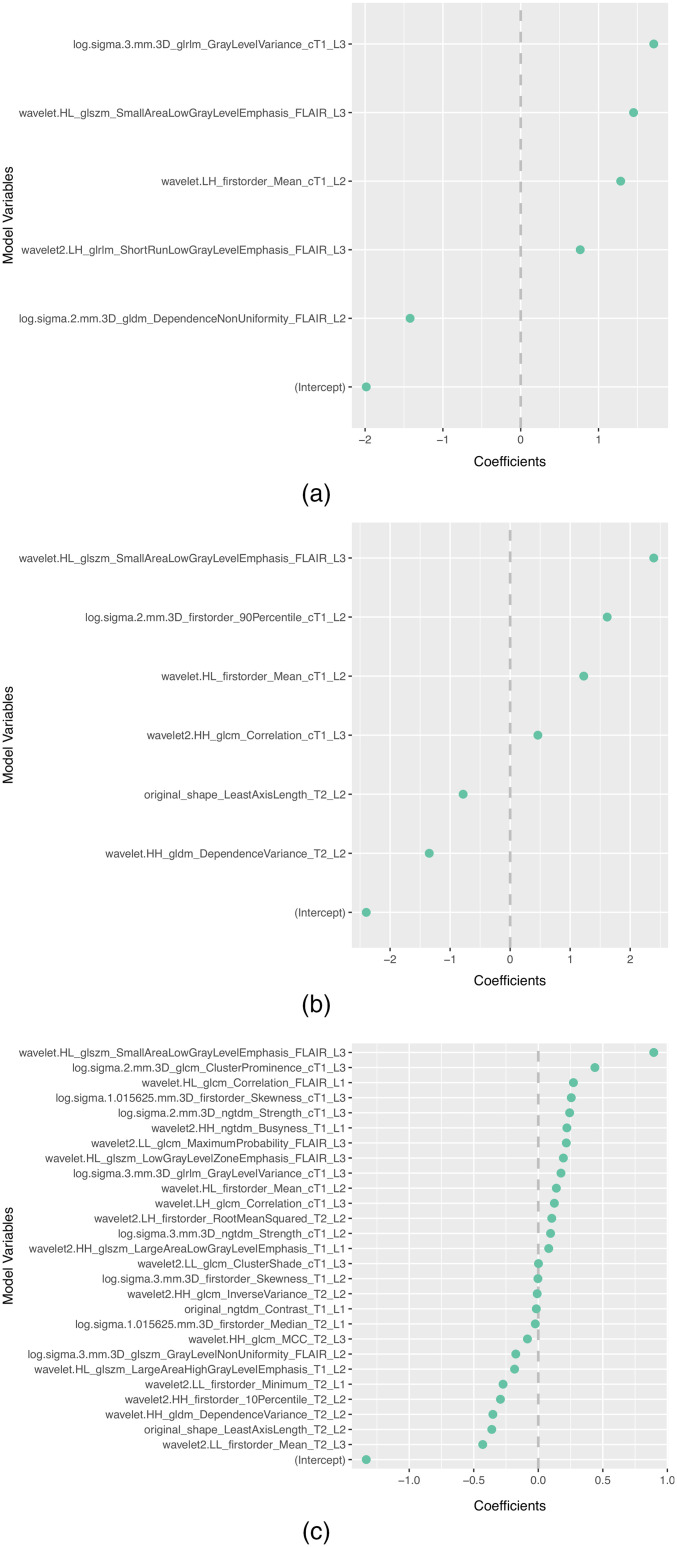
Representation of coefficients of selected features for models (a) A1, (b) A2, and (c) B when tuned with 70% of data from centers 1, 2, 3, and 4.

The subset of features selected more than once by the different feature selection methods in each scenario is presented in Table 8. In addition, the selected features and corresponding coefficients for the different models of both scenarios are shown in Appendix (Figs. 3–7). From these figures, it is possible to observe that the selection of robust and invariant features resulted in models A1 and A2 with consistently fewer features than model B.

**Table 8:** List of features selected by each method (A1, A2, and B) for each scenario (individual centers as independent test set or stratified partitioning of data from all four centers into training and test sets. Note: “3D” within features named log.sigma.< sigma_value>.mm.3D.< feature_class_feature_name> does not indicate the extraction of the feature in 3D but that the LoG is a 3D filter.

	Test 1			Test 2			Test 3			Test 4			Test mix			
	A1	A2	B	A1	A2	B	A1	A2	B	A1	A2	B	A1	A2	B	Total
wavelet.HL_firstorder_Mean_cT1_L2	1	1	0	1	1	1	0	0	0	0	0	1	0	1	1	8
wavelet.HH_gldm_DependenceVariance_T2_L2	1	0	0	1	1	1	0	0	0	0	0	0	0	1	1	6
wavelet.HL_glszm_SmallAreaLowGrayLevelEmphasis_FLAIR_L3	1	0	1	0	0	1	0	0	0	0	0	0	1	1	1	6
log.sigma.2.mm.3D_firstorder_90Percentile_cT1_L2	0	1	0	0	0	0	0	1	1	0	0	0	0	1	0	4
original_shape_MinorAxisLength_T1_L2	0	0	1	0	0	1	0	0	0	1	0	1	0	0	0	4
wavelet2.LL_firstorder_Mean_T2_L3	0	0	1	0	0	1	0	0	1	0	0	0	0	0	1	4
wavelet2.HL_glszm_LargeAreaHighGrayLevelEmphasis_T1_L2	0	0	0	0	0	1	1	1	1	0	0	0	0	0	0	4
wavelet.LH_gldm_LargeDependenceHighGrayLevelEmphasis_T1_L2	1	1	1	0	0	0	0	0	0	0	0	0	0	0	0	3
wavelet2.HH_glcm_Idm_T2_L2	0	0	1	0	0	0	0	0	0	1	0	1	0	0	0	3
original_firstorder_Mean_T2_L3	0	0	1	0	0	1	0	0	1	0	0	0	0	0	0	3
wavelet.HL_firstorder_10Percentile_T2_L2	0	0	1	0	0	1	0	0	1	0	0	0	0	0	0	3
wavelet2.LH_gldm_LowGrayLevelEmphasis_FLAIR_L3	0	0	1	1	0	1	0	0	0	0	0	0	0	0	0	3
log.sigma.3.mm.3D_firstorder_MeanAbsoluteDeviation_cT1_L3	0	0	1	0	0	0	1	0	1	0	0	0	0	0	0	3
wavelet.HL_firstorder_RootMeanSquared_cT1_L2	0	0	1	0	0	1	0	0	0	0	0	1	0	0	0	3
log.sigma.3.mm.3D_ngtdm_Strength_T2_L2	0	0	0	0	0	0	0	0	0	1	1	1	0	0	0	3
wavelet2.HH_glrlm_GrayLevelNonUniformity_T2_L2	1	1	0	0	0	0	0	0	0	0	0	0	0	0	0	2
wavelet2.HL_gldm_LargeDependenceHighGrayLevelEmphasis_T1_L2	1	0	1	0	0	0	0	0	0	0	0	0	0	0	0	2
wavelet.HL_glszm_GrayLevelNonUniformity_T2_L2	1	0	1	0	0	0	0	0	0	0	0	0	0	0	0	2
log.sigma.1.015625.mm.3D_firstorder_Mean_T2_L1	0	0	1	0	0	1	0	0	0	0	0	0	0	0	0	2
wavelet2.HH_gldm_LargeDependenceLowGrayLevelEmphasis_T1_L2	0	0	1	0	0	0	0	0	1	0	0	0	0	0	0	2
wavelet2.HL_gldm_LargeDependenceLowGrayLevelEmphasis_T1_L2	0	0	1	0	0	0	0	0	1	0	0	0	0	0	0	2
wavelet.HH_glcm_SumEntropy_T2_L2	0	0	1	0	0	1	0	0	0	0	0	0	0	0	0	2
wavelet2.LL_glcm_MCC_T2_L2	0	0	1	0	0	1	0	0	0	0	0	0	0	0	0	2
wavelet.LH_glcm_Correlation_cT1_L3	0	0	1	0	0	0	0	0	0	0	0	0	0	0	1	2
wavelet2.HL_glszm_ZoneEntropy_T1_L2	0	0	0	1	0	1	0	0	0	0	0	0	0	0	0	2
wavelet.HL_glszm_ZoneEntropy_T1_L2	0	0	0	0	1	0	0	1	0	0	0	0	0	0	0	2
wavelet2.HH_glcm_InverseVariance_T2_L2	0	0	0	0	0	1	0	0	0	0	0	0	0	0	1	2
wavelet2.HH_glszm_LargeAreaLowGrayLevelEmphasis_T1_L1	0	0	0	0	0	1	0	0	0	0	0	0	0	0	1	2
wavelet2.LL_glcm_MaximumProbability_FLAIR_L3	0	0	0	0	0	1	0	0	0	0	0	0	0	0	1	2
wavelet2.LL_glcm_ClusterShade_cT1_L3	0	0	0	0	0	1	0	0	0	0	0	0	0	0	1	2
wavelet.HL_gldm_DependenceVariance_FLAIR_L3	0	0	0	0	0	0	1	0	1	0	0	0	0	0	0	2
wavelet.LH_glszm_SizeZoneNonUniformity_FLAIR_L1	0	0	0	0	0	0	1	0	1	0	0	0	0	0	0	2
wavelet.HL_glszm_LargeAreaHighGrayLevelEmphasis_T1_L2	0	0	0	0	0	0	0	1	0	0	0	0	0	0	1	2
wavelet.HL_glcm_Correlation_FLAIR_L1	0	0	0	0	0	0	0	0	1	0	0	0	0	0	1	2
wavelet2.HH_firstorder_10Percentile_T2_L2	0	0	0	0	0	0	0	0	0	1	0	0	0	0	1	2
log.sigma.1.015625.mm.3D_ngtdm_Contrast_T2_L2	0	0	0	0	0	0	0	0	0	1	0	1	0	0	0	2
log.sigma.3.mm.3D_gldm_LargeDependenceLowGrayLevelEmphasis_FLAIR_L3	0	0	0	0	0	0	0	0	0	1	0	1	0	0	0	2
wavelet2.HL_glcm_ClusterShade_cT1_L3	0	0	0	0	0	0	0	0	0	0	1	1	0	0	0	2
log.sigma.1.015625.mm.3D_glrlm_ShortRunLowGrayLevelEmphasis_T1_L3	0	0	0	0	0	0	0	0	0	0	1	1	0	0	0	2
log.sigma.3.mm.3D_glrlm_GrayLevelVariance_cT1_L3	0	0	0	0	0	0	0	0	0	0	0	0	1	0	1	2
original_shape_LeastAxisLength_T2_L2	0	0	0	0	0	0	0	0	0	0	0	0	0	1	1	2

## 4 Discussion and Conclusions

The use of radiomics and the discovery of associations between radiomic features and genomic characteristics has the potential to make the diagnosis and stratification using important genomic biomarkers accessible to a larger patient population. However, being a high-dimensional problem, where the number of patients is much lower than the number of variables, unstable solutions are very likely to be found. Furthermore, brain tumor diagnosis, surgery/radiotherapy planning, and treatment assessment rely on MRI, which is highly dependent on the scanner, sequence, and sequence parameters, where even parametric maps suffer from vendor/operator-specific variability, hindering the performance and generalizability of the developed models.

In this study, we hypothesize that the method developed by Peters et al.^3^ to find variables that are part of causal structures, based on their invariance to different environments, may also be used to find robust and invariant features that lead to smaller and more generalizable models. This method takes advantage of data from multiple hospitals, various scanners, and images acquired with different sequence parameters. This hypothesis was assessed using two different scenarios commonly found in the literature: validation on an independent held-out test set and a held-out test set obtained through partitioning of the whole dataset.

Table 3 shows that models using the two variants of the proposed feature selection method have high performance on the cross-validation and dropped their performance on the test set. In the case in which center 2 was used as the held-out test set, all models show poor generalizability and robustness to data outside of the distribution they were trained with, and none of the models were able to classify the unique IDH1/2 wild-type case correctly. When using centers 1 and 3 as independent held-out test sets, models B showed an increase in sensitivity but a drop in specificity, when compared with their cross-validation performance. Model B on center 4 as the independent held-out test set misclassified all 15 IDH1/2 wild-type cases.

In summary, all models showed similar performance for center 1 as the independent held-out test set and showed very poor performance for center 2 as the independent held-out test set. As for the case in which center 3 was used as the held-out test set, models A1 and B show equal sensitivity, but model B yielded a slightly better specificity (0.79 [0.61; 0.90] versus 0.72 [0.54; 0.85]), leading to better accuracy and MCC, although the corresponding CIs of both models for these metrics present a considerable overlap. When the data from center 4 was used as the independent held-out test set, model A1 attained the best performance (higher sensitivity and lower specificity), followed by model A2 and, finally, B, both with equal specificity but with model B presenting a 0 sensitivity. Although, the CIs of sensitivity of models A1 and A2 and of AUC, accuracy, and specificity of models A1, A2, and B show overlap, the CI of the balanced measure MCC was higher for model A1 without overlap with the CIs of models A2 and B.

Despite these results, none of the models showed an accuracy statistically higher than the NIR. The comparison between AUCs of the different models also did not show statistically significant differences (p-values>0.05). Across the held-out test sets, the performance was significantly better than random guessing for model A2 on center 2, models A1 and B on center 3, and model A1 on center 4. In these cases, this may be a result of the relatively small number of cases.

When analyzing the cross-validation and held-out test results obtained using models tuned using 70% of the whole data set comprising the four centers and assessed on the remaining 30%, it is possible to observe that, between cross-validation and the held-out test performance metrics, model A1 showed inferior performance for all metrics and model B shows lower sensitivity but slightly higher specificity, being reflected in other metrics such as AUC and MCC. Nonetheless, model A2 showed very similar performance between the assessment on the held-out test set and the cross-validation performance, with only the AUC falling outside the cross-validation’s 95% CI. As for the comparison of held-out test performance across models, model A2 attained higher performance than models A1 and B, but overlap was observed between all performance metrics’ CIs with the exception of MCC for model A2, which was higher. In this scenario, significant differences were not found between the models’ AUCs (p-values>0.05). Still, all models were significantly better than random guessing, and the accuracy of model A2 was significantly greater than the NIR.

Considering the results of both scenarios, the proposed methods attained a relatively good performance when compared with the traditional approach, in almost all studied cases, except for center 2 as the held-out test set, where all models showed poor performance, and on center 3 as the held-out test set, where model A1 showed equal specificity but lower sensitivity.

To better understand why each of the two feature selection methods performed better on each scenario, additional data may be needed to make any claims. However, these may happen because, in scenario 1, the assessment is performed on out-of-distribution data and only three centers are used for feature selection and model tuning, whereas in scenario 2 all four centers were used for feature selection and model tuning, and the testing was performed in similar data.

Furthermore, from Figs. 3–7, it is possible to observe that models A1 and A2 achieved the reported performance with much fewer features than model B. In some cases, models A1 and A2 comprise ∼1/4 to ∼1/5 of the number of features in model B. By reducing the number of features within a model, two critical aspects of ML algorithms such as explainability and the investigation of misclassifications in error analysis are improved.

When analyzing the frequency of the selection of features in Table 8, it is possible to observe that the majority of the features that are selected more than once are wavelet-based, followed by LoG-based, and, finally, original features. The feature with the most occurrences (8) was wavelet HL first-order mean extracted from the necrotic region segmentation in the contrast-enhanced T1w images. For example, a more in-depth analysis of this feature’s selection in scenario 1 shows that it was not picked by the proposed methods for the cases of centers 3 and 4 as the held-out test sets. A possible explanation may be the large number of cases of both centers when compared with the remaining centers. This may bias the selection when both centers are included but lacks support when one of these is not utilized in the feature selection.

Several studies have investigated the use of radiomics to predict IDH1/2 mutation status in low-grade, high-grade, or mixed glioma patient cohorts using basic structural and advanced multiparametric MRI.^23^^–^^29^ Although the overall performance of the feature selection herein proposed showed good performance compared with studies performed only in low-grade^23^^–^^26^ or high-grade^27^ glioma patient cohorts, we will focus our comparison in studies with mixed cohorts.^28^^,^^29^ We will use the performance of model A2 in the second scenario for the comparisons, as these studies did not use independent held-out test sets as in scenario 1. The first study, by Wu et al.,^28^ assessed models using several types of classifiers. Their best model, random forest, yielded an AUC of 0.93 and an accuracy of 0.89, which is higher than the performance of the proposed method. However, due to the behavior of AUC and accuracy in the case of imbalanced sets and without additional metrics, a more thorough comparison is not possible. In Sudre et al.,^29^ the authors assessed, using repeated cross-validation, a random forest model with radiomic features extracted from advanced multiparametric MRI, dynamic susceptibility contrast-MRI (DSC-MRI), acquired in six different centers. Their model yielded an accuracy of 0.71, a sensitivity of 0.65, and a specificity of 0.77. A comparison with the method proposed in our study reveals that it can improve performance even when compared with features extracted from more advanced multiparametric MRI, despite the link between DSC-MRI perfusion biomarkers and tumor vascularity and possible IDH role in angiogenesis.

The method herein proposed shows the potential to improve the performance and generalizability of models, leading in some cases to better performance than what has been reported in the literature.

This study has several limitations. The study was performed with a relatively small data set, and no prospective data were used. In addition, only four environments (corresponding to the number of centers contributing with MRI images) were considered. Future studies with larger cohorts including of a large number of environments and a large number of cases per environment are required to confirm the good results of the methodology herein proposed. In theory, such increases of the number of environments and the number of cases per environment may also result in an improvement of the observed performance, promoted by the selection of more robust features and more generalizable models.

In this study, we assessed the use of a method that promotes the selection of predictive features invariant to the acquisition environment, such as different scanners and/or sequence parameters. This method, in its two variants, was compared with a more traditional and widely used method, having achieved good performances, both in terms of robustness and generalizability, with significantly fewer features in the majority of the scenarios used for their evaluation.

## 5 Appendix

### 5.1 Weights and Features of Models

Figures 3–7 show the features and corresponding weights in the models obtained using the different feature selection methods (A1, A2, and B) for the different scenarios (center 1 as held-out test set, center 2 as held-out test set, center 3 as held-out test set, and center 4 as held-out test set, stratified partitioning of data from all four centers, respectively).

## Code, Data, and Materials Availability

The radiomics files used in the analysis, parameter files used for the feature extraction, and analysis source code are available at https://github.com/JoaoSantinha/Radiogenomics_Gliomas_Pilot.
